# Clinicopathological characteristics and its association with digestive system tumors of 1111 patients with *Schistosomiasis japonica*

**DOI:** 10.1038/s41598-023-42456-9

**Published:** 2023-09-13

**Authors:** Yang Yang, Xiao-Yi Wang, Chun Duan, Zi-Jian Wang, Hao-Yu Sheng, Xiu-Liang Xu, Wen-Jie Wang, Jiang-Hua Yang

**Affiliations:** 1 Department of Infectious Diseases, Yijishan Hospital of Wannan Medical College, Wuhu, Anhui People’s Republic of China; 2 Class1 Grade 2019, Department of Stomatology, Bengbu Medical College, Bengbu, Anhui People’s Republic of China; 3 Department of Infectious Diseases, The People’s Hospital of Chizhou, Chizhou, Anhui People’s Republic of China

**Keywords:** Diseases, Medical research

## Abstract

*Schistosomiasis japonicum* can cause different degrees of organ damage and complex human immune pathological reactions, which often invade the intestine and liver. The purpose of this study was to explore the pathological types and pathological changes of *Schistosomiasis* and their correlation with some digestive system tumors. Hematoxylin eosin staining was performed on the diseased tissues of 1111 *Schistosomiasis* cases. We counted the deposition sites of *Schistosoma* eggs, analyzed the pathological characteristics, and compared the clinicopathological characteristics of *Schistosomiasis* associated digestive system tumors and non-*Schistosomiasis* digestive system tumors. We found that *Schistosoma japonicum* can cause multi organ and multi system damage, with 469 cases of inflammation, 47 cases of adenoma, and 519 cases of adenocarcinoma. Other types include cysts, stromal tumors, malignant lymphomas, and neuroendocrine tumors. *Schistosomiasis* associated tumors, including gastric cancer, liver cancer, colon cancer and rectal cancer, were compared with non-*Schistosomiasis* tumors. There were significant differences in age, gender and tumor differentiation between the two groups. Our study shows *Schistosomiasis* is a systemic disease, causing multiple organ and system damage in the human body. Its clinicopathological types are diverse, and there may be a pathological change process of “Inflammation-adenoma-carcinoma”. *Schistosomiasis* associated digestive system tumors differ from non-*Schistosomiasis* tumors in some clinicopathological features.

## Introduction

*Schistosomiasis* is a zoonotic parasitic disease. In China, *Schistosoma japonicum* is mainly prevalent in the Yangtze River basin^1^. By the end of 2021, there were 12 *Schistosomiasis* endemic provinces (municipalities and autonomous regions) and 450 *Schistosomiasis* endemic counties (cities and districts). There are approximately 72,937 cases of *Schistosomiasis* and 29,037 cases of advanced *Schistosomiasis* in China. In the life cycle of *Schistosoma japonicum*, the tail spider, the adult worm and the egg can all lead to varying degrees of damage and complex immunopathological reactions to the human body^2^. *Schistosomiasis* is a chronic immune disease caused by egg granuloma. Pathological examination is one of the important methods for the diagnosis of *Schistosomiasis*^3^. Its pathological basis is the stimulation of a large number of mature eggs deposited in the intestinal and liver tissues, the pathological immune response of the host to the soluble antigen secreted by live cercariae, and the formation of egg granuloma and secondary fibrosis^4^. Rarely known by people, the eggs can also cause different types of pathological changes in other parts of the body. However, only a few case reports and small sample size studies can not reflect the correlation between *Schistosomiasis* and the pathological changes in other body parts.

With the development of society and the changes of people’s lifestyle, the incidence and mortality of cancer are rising rapidly^5^, among which digestive tract-related malignant cancer have become the main cause of human death. In recent years, the relationship between *Schistosomiasis* and tumor attracts much attention. According to the International Agency for Research on Cancer (IARC), *Schistosoma haematobium* has a clear carcinogenic effect on humans. *Schistosoma haematobium* can cause bladder cancer. Its pathogenic process is that the adults lay eggs in the bladder venous plexus, and the eggs spread along the blood vessels to the bladder, ureter and genital tract. The eggs stimulate the tissue to produce continuous inflammatory response, leading to *Urogenital Schistosomiasis* (UGS)^6, 7^. Bladder cancer is one of the common complications of UGS. Although the carcinogenic effects of *Schistosoma japonicum* and *Schistosoma mansoni* are not clear, they are often found parasitic in mesenteric veins and oviposit. The eggs spread with the blood. Eggs deposited in liver and intestinal tissues recruit a variety of inflammatory factors leading to granuloma formation. Some of these inflammatory cells can release a large amount of reactive oxygen species and NO, leading to oxidative stress-induced DNA damage, thereby causing malignant transformation of tissue cells^8–11^. In China, where *Schistosomiasis japonica* is endemic, gastric cancer, liver cancer and colorectal cancer are common malignant tumors of the digestive system. In this study, 1111 cases of *Schistosoma japonicum* were pathologically confirmed to observe the deposition site and pathological types of *Schistosoma* eggs, and to compare the pathological features of *Schistosomiasis* associated tumors and non-*Schistosomiasis* tumors.

## Materials and methods

### Patient data collection

The clinicopathological data of 1111 patients with chronic *Schistosomiasis* in the First Affiliated Hospital of Wannan Medical College and Chizhou people’s Hospital from January 2015 to December 2021 were collected and retrospectively in this study. (These two hospitals are the main hospitals for treating *Schistosomiasis* patients, and a total of 2500 beds are opened to treat patients in *Schistosomiasis* endemic areas). The patients included in the study were all from the *Schistosomiasis* endemic area in southern Anhui Province. The included patients showed *Schistosoma* eggs by histopathological examination of lesions, and patients with other parasitic infections and overseas tourism history were excluded. Pathological specimens were obtained by surgical resection, endoscopic biopsy, and needle biopsy. All pathological specimens were diagnosed by two pathologists.

A total of 498 patients with gastric cancer, liver cancer, colon cancer and rectal cancer were selected from this study and included in *Schistosomiasis* associated tumor group. The *Schistosoma* eggs and tumor cells could be seen in the pathological examination of the included patients, and the tumor and metastatic tumor caused by other factors (such as bacterial infection, viral infection, poison contact, genetic and other factors) were excluded. We collected almost the same number of patients with non-*Schistosomiasis* tumors each year. A total of 569 patients with gastric cancer, liver cancer, colon cancer and rectal cancer without *Schistosomiasis* in the same region during the same period were collected as non-*Schistosomiasis* tumor group. These patients were definitely diagnosed by clinicopathology. Metastatic tumors and tumors caused by other factors were excluded from the enrolled patients. All tumor patients were confirmed by clinicopathology. The clinicopathological data of the two groups were collected.

This study was approved by the ethics committee of the First Affiliated Hospital of Wannan Medical College [Research Proposal Notification IRB Review Decision (2022) (No.33)]. Informed consent was obtained from all patients which were involved in this study. All procedures performed in studies involving human participants were in accordance with the ethical standards of the institutional and national research committee and with the Declaration of Helsinki.

### Hematoxylin–eosin staining

1218 pathological specimens from 1111 patients with *Schistosomiasis* were roasted in an oven at 60 °C for 30 min, and after taking out the specimen, put it into xylene I and II for dewaxing for 5 min each. The specimens were put into gradient alcohol 100%, 95%, 85% and 75% for 1 min respectively for hydration. Then all specimens were rinsed with distilled water for 1 min, stained with hematoxylin for 4 min, differentiated with hydrochloric acid and ethanol for 15 s after rinsing, and then rinsed with pure water and then added with 0.5% eosin staining solution for 1 min. The specimens were rinsed with distilled water and dried, then put into gradient alcohol 95% I, 95% II, 100% I, 100% II for 5 min each, finally put into xylene I and II for 5 min each, mount.

### Statistical method

SPSS 20.0 statistical software was used to analyze the data. Data are presented as frequency (n), percentage (%), mean ± standard deviation (SD), and quartiles, respectively. The clinicopathological data of *Schistosomiasis* associated tumor group and non-*Schistosomiasis* tumor group were analyzed (Variables such as age, sex, and pathological characteristics of tumors were analyzed). Student t test and Mann Whitney U test were used. Categorical variables were analyzed using chi square test. *P* < 0.05 was statistically significant.

## Result

### Distribution characteristics of *Schistosomiasis* patients

**Figure 1:**
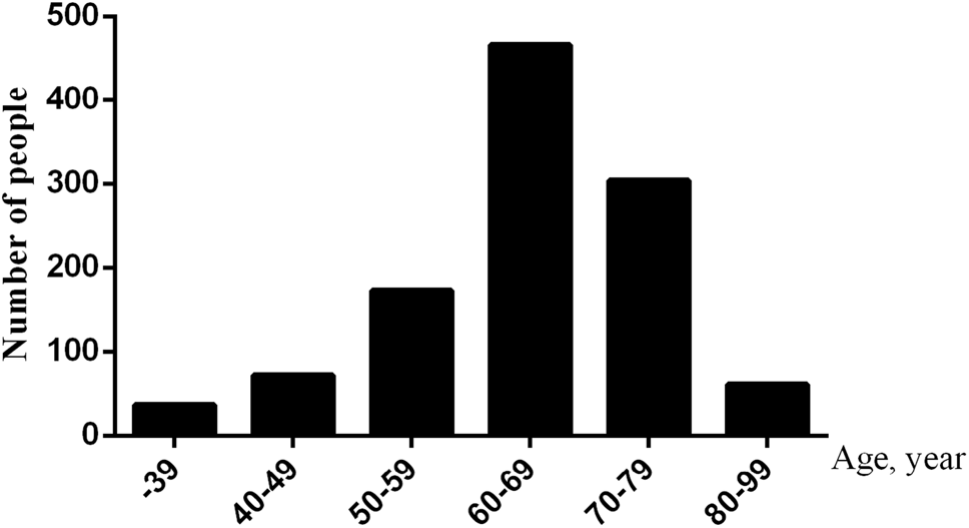
Age distribution of patients with *Schistosomiasis.*

A total of 1111 cases of *Schistosomiasis* were collected from January 2015 to December 2021, including 747 males and 364 females, and aged from 18 to 93 years. There are 36 cases (4.05%) from 10 to 39 years old, 72 cases (6.481%) from 40 to 49 years old, 173 cases (15.572%) from 50 to 59 years old, 465 cases (41.855%) from 60 to 69 years old, 304 cases (27.363%) from 70 to 79 years old and 61 cases (5.491%) from 80 to 99 years old. The incidence of *Schistosomiasis* is mainly concentrated in the middle-aged and elderly people aged 60–69 years. (Fig. 1).

### Deposition of *Schistosoma* eggs

**Figure 2:**
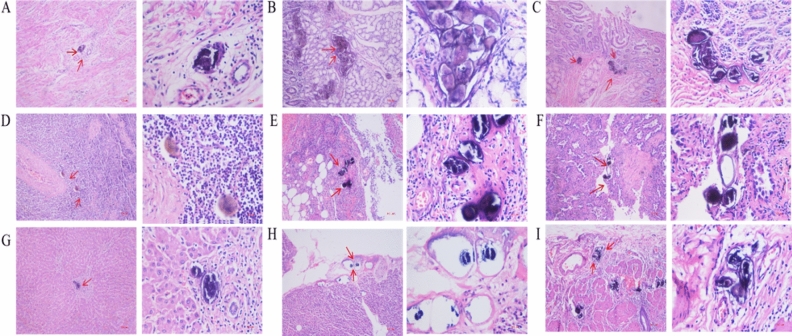
*Schistosoma* eggs deposition in the organs of the digestive system. (**A**) *Schistosoma* eggs are deposited in esophageal tissue; (**B**) *Schistosoma* eggs are deposited in the stomach tissue; (**C**) *Schistosoma* eggs are deposited in duodenal tissue; (**D**) *Schistosoma* eggs are deposited in colon tissue; (**E**) *Schistosoma* eggs are deposited in appendix tissue; (**F**) *Schistosoma* eggs are deposited in rectal tissue; (**G**) *Schistosoma* eggs are deposited in liver tissue; (**H**) *Schistosoma* eggs are deposited in pancreatic tissue; (**I**) *Schistosoma* eggs are deposited in gallbladder tissue. Magnification, × 100 or × 400.

**Figure 3:**
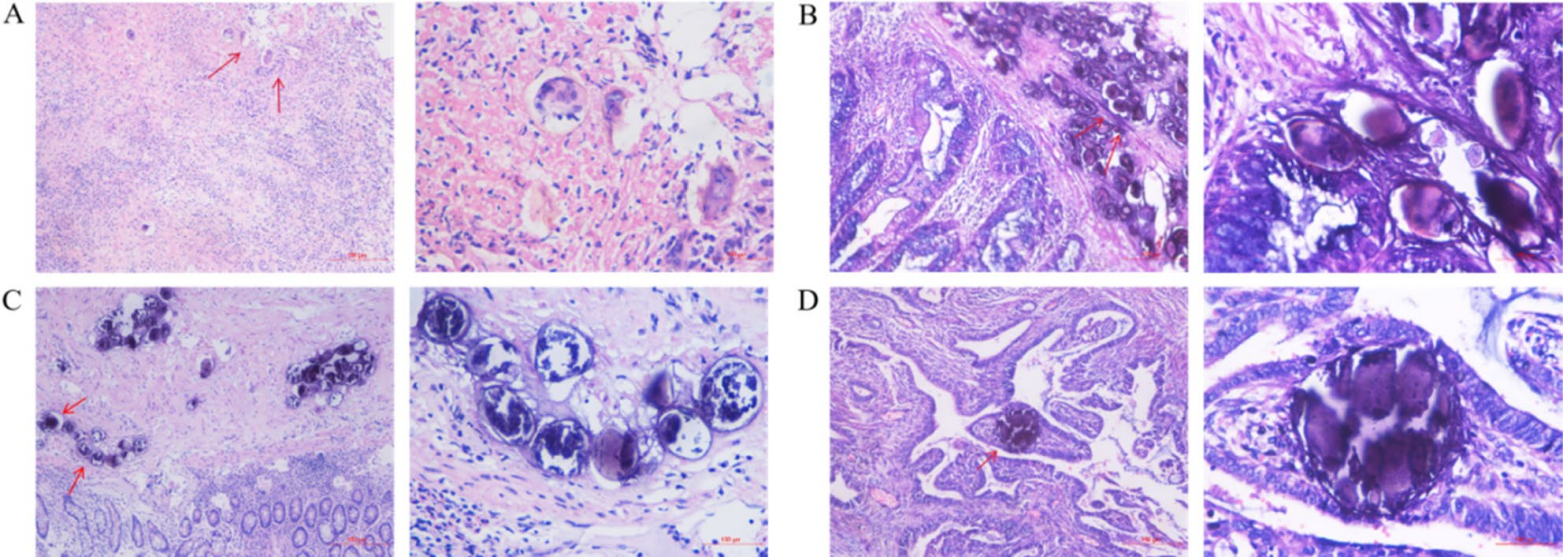
*Schistosoma* eggs deposition in the other organs. (**A**) *Schistosoma* eggs are deposited in brain tissue; (**B**) *Schistosoma* eggs are deposited in uterine tissue; (**C**) *Schistosoma* eggs are deposited in ovarian tissue; (**D**) *Schistosoma* eggs are deposited in lung tissue. Magnification, × 100 or × 400.

Analysis of 1218 *Schistosoma* eggs deposition organs from 1111 patients revealed that *Schistosoma* eggs were mainly distributed in the digestive system, accounting for up to 84.56%. The main deposition organs of *Schistosoma* eggs are colon, rectum, appendix and stomach, and a few eggs are found in rare deposition organs such as esophagus, duodenum, ileum, cecum, gallbladder and pancreas. The egg deposition rate of *Schistosoma japonicum* in the lymphatic system was 13.55%, second only to the digestive system. In addition, *Schistosoma* eggs were also deposited in the reproductive system (1.07%), respiratory system (0.58%), nervous system (0.25%), etc. (Table 1; Figs. 2, 3).

**Table 1:** Distribution of egg deposition sites of *Schistosoma japonicum.*

System	Organs	Number of persons	Percentage
Digestive system (84.56%)	Esophagus	2	0.164
	Stomach	177	14.532
	Duodenum	3	0.246
	Ileum	2	0.164
	Cecum	2	0.164
	Colon	244	20.033
	Appendix	278	22.824
	Rectum	174	14.286
	Liver	121	9.934
	Pancreas	8	0.657
	Gall bladder	19	1.560
Nervous system (0.25%)	Brain	3	0.246
Reproductive system (1.07%)	Uterus	1	0.082
	Ovary	8	0.657
	Fallopian tubes	4	0.328
Respiratory system (0.58%)	Lung	7	0.575
Lymphatic system (13.55%)	Lymph nodes	165	13.547

### Pathological features

In addition to the lymph nodes with *Schistosoma* egg deposits (165 cases), the pathological types of 1053 organ lesions with *Schistosoma* egg deposits are mainly inflammation, adenoma and adenocarcinoma, and only a few are other pathological types, such as cysts, stromal tumors, malignant lymphomas and neuroendocrine tumors. 469 organ lesions were inflammatory, including stomach, duodenum, small intestine (ileum, cecum), colon, appendix, rectum, liver, gallbladder, brain, ovary, fallopian tube, lung, and the appendix was the most prone to inflammation. 47 organ lesions were adenomas, including stomach, duodenum, colon, appendix, rectum, gallbladder, ovary, and colon was prone to adenomas. 519 organ lesions were adenocarcinoma, including esophagus, stomach, duodenum, ileum, cecum, colon, rectum, liver, pancreas, gallbladder, ovary, lung, and colon, rectum, stomach were prone to adenocarcinoma. Inflammation, adenoma, and adenocarcinoma can occur simultaneously in the stomach, duodenum, colon, rectum, gallbladder, and ovary. (Table 2).

**Table 2:** Pathological types of egg-related diseases of *Schistosoma japonicum.*

Organs	Inflammation	Adenoma	Adenocarcinoma	Others	Total
Esophagus			2		2
Stomach	11	7	156	3^a^	177
Duodenum	1	1	1		3
Ileum	1		1		2
Cecum	1		1		2
Colon	62	21	158	3^b^	244
Appendix	269	8		1^c^	278
Rectum	26	7	140	1^d^	174
Liver	69		44	8^e^	121
Pancreas			7	1^f^	8
Gall bladder	13	2	4		19
Brain	3				3
Uterus				1^g^	1
Ovary	4	1	3		8
Fallopian tubes	4				4
Lung	5		2		7

Significant values are in a–g. ^a^:3 cases of gastric stromal tumor; ^b^:2 cases of colonic malignant lymphoma and 1 case of colonic malignant stromal tumor; ^c^:1 case of appendiceal neuroendocrine tumor; ^d^:1 case of rectal neuroendocrine tumor; ^e^:8 cases of liver cyst; ^f^:1 case of pancreatic cyst; ^g^:1 case of uterine cyst.

The ages of 1035 cases with inflammation, adenoma and adenocarcinoma were (61.555 ± 13.025) years, (63.936 ± 9.628) years and (66.730 ± 9.156) years, respectively. There was no significant difference in age between the inflammation group and the adenoma group (*P* = 0.087), but there was a significant difference in age between the inflammation group and the adenocarcinoma group (*P* < 0.001), and there was a significant difference in age between the adenoma group and the adenocarcinoma group (*P* = 0.026). There were 664 males and 371 females, and the difference was statistically significant (*P* = 0.047). (Table 3).

**Table 3:** Comparison of gender and age of patients with different pathological types.

	Male, n	Female, n	Age, year
Inflammation	292	177	61.555 ± 13.025
Adenoma	24	23	63.936 ± 9.628
Adenocarcinoma	348	171	66.730 ± 9.156^a^

a Comparison between adenoma group and adenocarcinoma group (*P* = 0.026).

### Clinicopathological analysis of *Schistosomiasis* associated tumor group and non-*Schistosomiasis* tumor group

Based on this group of data, we selected the tumors with the top four proportions of *Schistosomiasis* combined with adenocarcinoma, gastric cancer, liver cancer, colon and rectal cancer. *Schistosomiasis* associated tumors were compared with non-*Schistosomiasis* tumors in the same period.

Comparison of clinicopathological data of gastric cancer:

In the gastric cancer group, the average ages of *Schistosomiasis* associate gastric cancer group (n = 156) and non-*Schistosomiasis* gastric cancer group (n = 179) were (67.449 ± 7.959) years and (62.14 ± 10.832) years, respectively, and the ages of the former were significantly higher than those of the latter (*P* < 0.001). There were more males than females in both groups, but the male to female ratio in the *Schistosomiasis* associated gastric cancer group was significantly higher than that in the non-*Schistosomiasis* gastric cancer group (*P* = 0.006). In terms of pathological characteristics of tumors in the two groups, *Schistosomiasis* associated gastric cancer mainly occurred in the body of the stomach, while non-*Schistosomiasis* gastric cancer mainly occurred in the pylorus, and the difference was statistically significant (*P* = 0.001). The differentiation degree of tumors in the two groups was different (*P* = 0.003), tumor appearance was different in 214 cases (*P* = 0.745), tumor blood vessels were different in 215 cases (*P* = 0.116), and regional lymph node metastasis was different in 214 cases (*P* = 0.904). (Table 4).

**Table 4:** Comparison of clinicopathological features of patients with gastric cancer.

Variable	With *Schistosomiasis* n = 156	Without *Schistosomiasis* n = 179	*P*-value
Age, mean ± standard deviation, years	67.449 ± 7.959	62.145 ± 10.832	< 0.001
Sex			0.006
Male	132	129	
Female	24	50	
Location			0.001
Cardia and fundus of stomach	35	43	
Gastric body	52	28	
Pylorus	69	108	
Phenotype			0.745
Ulcer type	94	112	
Infiltrating	21	27	
Depressed	24	25	
Flat type	6	8	
Raised type	11	7	
Differentiation			0.003
Poor	100	109	
Moderate	51	46	
Well	5	24	
Cancer thrombus			0.113
Yes	57	51	
No	99	128	
Lymph node metastasis			0.904
Yes	69	78	
No	87	101	

### Comparison of clinicopathological data of hepatocellular carcinoma

In patients with liver cancer, the average ages of *Schistosomiasis* associated liver cancer group (n = 44) and non-*Schistosomiasis* liver cancer group (n = 64) were (65.886 ± 6.738) years and (64.438 ± 9.179) years, respectively, and there was no significant difference in age between the two groups (*P* = 0.43). There were more males than females in the two groups, but there was no statistical difference in the proportion of males and females in the two groups (*P* = 0.58). The proportion of poorly differentiated tumors in the *Schistosomiasis* associated liver cancer group was significantly higher than that in the non-*Schistosomiasis* liver cancer group (*P* = 0.019), but there was no statistical difference in the source of tumor cells (*P* = 0.135), the number of tumors (*P* = 0.159), the presence of tumor thrombus (*P* = 0.4) and the integrity of tumor envelope (*P* = 0.077). (Table 5).

**Table 5:** Comparison of pathological features of patients with liver cancer.

Variable	With *Schistosomiasis* n = 44	Without *Schistosomiasis* n = 64	*P*-value
Age, mean ± standard deviation, years	65.886 ± 6.738	64.438 ± 9.179	0.43
Sex			0.58
Male	28	44	
Female	16	20	
Cell source			0.135
Hepatocyte	33	36	
Bile duct cells	10	26	
Mixed cell	1	2	
Differentiation			0.019
Poor	19	12	
Moderate	19	36	
Well	6	16	
Number of tumors			0.159
Single	39	50	
Multiple	5	14	
Cancer thrombus			0.4
Yes	15	17	
No	29	47	
Envelope integrity			0.077
Yes	30	53	
No	14	11	

### Comparison of clinicopathological data of colon cancer

Among colon cancer patients, the average ages of *Schistosomiasis* associated colon cancer group (n = 158) and non-*Schistosomiasis* colon cancer group (n = 159) were (67.331 ± 10.082) years and (61.194 ± 11.675) years, respectively, with statistical differences (*P* < 0.001). There were more males than females in the two groups, and the male to female ratio in the *Schistosomiasis* associated colon cancer group was significantly higher than that in the non-*Schistosomiasis* colon cancer group (*P* = 0.005). In terms of tumor pathological characteristics, there was a statistically significant difference in the degree of differentiation between the two groups (*P* = 0.013), but there was no difference in the tumor prone site (0.177), tumor phenotype (0.195), presence or absence of tumor thrombus (0.069), and presence or absence of lymph node metastasis (0.076). (Table 6).

**Table 6:** Comparison of pathological features of patients with colon cancer.

Variable	With *Schistosomiasis* n = 158	Without *Schistosomiasis* n = 159	*P*-value
Age, mean ± standard deviation, years	67.331 ± 10.082	61.194 ± 11.675	< 0.001
Sex			0.005
Male	108	84	
Female	50	75	
Location			0.177
Sigmoid colon	54	58	
Descending colon	11	13	
Transverse colon	7	14	
Ascending colon	22	28	
Ileocecal	64	46	
Phenotype			0.195
Ulcer type	109	96	
Infiltrating	3	1	
Constricted	1	3	
Raised type	45	59	
Differentiation			0.013
Poor	38	35	
Moderate	108	86	
Well	12	28	
Cancer thrombus			0.069
Yes	11	8	
No	44	52	
Lymph node metastasis			0.076
Yes	18	25	
No	37	35	

### Comparison of clinicopathological data of rectal cancer

In patients with rectal cancer, the average ages of *Schistosomiasis* associated rectal cancer group (n = 140) and non-*Schistosomiasis* rectal cancer group (n = 167) were (65.706 ± 9.5549) years and (59.517 ± 15.242) years, respectively, with statistically significant differences (*P* < 0.001). There were more males than females in the two groups, and the male to female ratio of *Schistosomiasis* associated rectal cancer was higher than that of non-*Schistosomiasis* rectal cancer group, but the difference was not statistically significant (*P* = 0.061). In terms of tumor pathological characteristics, there was a statistically significant difference in tumor differentiation between the two groups (*P* = 0.001), but there was no statistically significant difference in tumor phenotype (*P* = 0.378), tumor thrombus (*P* = 0.388), and lymph node metastasis (*P* = 0.331). (Table 7).

**Table 7:** Comparison of pathological features of patients with rectal cancer.

Variable	With *Schistosomiasis* n = 140	Without *Schistosomiasis* n = 167	*P*-value
Age, mean ± standard deviation, years	65.706 ± 9.554	59.517 ± 15.242	< 0.001
Sex			0.061
Male	99	101	
Female	41	66	
Phenotype			0.378
Ulcer type	97	126	
Infiltrating	3	5	
Constricted	0	1	
Flat type	1	0	
Raised type	39	35	
Differentiation			0.001
Poor	25	23	
Moderate	107	109	
W ell	8	35	
Cancer thrombus			0.388
Yes	27	39	
No	113	128	
Lymph node metastasis			0.331
Yes	24	36	
No	116	131	

## Discussion

*Schistosomiasis* is the most important parasitic disease that endangers people’s health^12, 13^. There are mainly six kinds of *Schistosomiasis* that infect humans: *Schistosoma haematobium*, *Schistosoma mansoni*, *Schistosoma japonicum*, *Schistosoma mekongi*, *Schistosoma interdental* and *Schistosoma malayi*^14^. China is an endemic area of *Schistosomiasis japonica*. *Schistosomiasis* is prevalent in the Yangtze River Basin and 12 provinces in the south of China. The pathological changes of *Schistosomiasis* are mainly caused by eggs, which are mainly deposited in the liver and colon wall of the host. Granuloma and fibrosis caused by *Schistosomiasis* are the main pathological changes of *Schistosomiasis*^15^. The formation of egg granuloma is an immune response of the host to pathogenic factors. On the one hand, the eggs are destroyed and removed by granuloma reaction, and the antigens released by eggs can be isolated and removed. Reduce the formation of antigen–antibody complex in blood circulation and damage to the body. On the other hand, granuloma reaction destroys the normal tissue of the host, and the continuous formation of egg granuloma forms interconnected scars, leading to a series of lesions such as trunk cirrhosis and intestinal wall fibrosis^16^.

*Schistosoma japonicum* usually lives in the portal vein system, mainly involving the colon and liver^17^, especially the sigmoid colon and rectum, and small intestinal lesions are rare^18^. Occasionally, in some cases of severe infection, adult worms and egg granulomas are found outside the portal system, causing ectopic damage, mainly in the lungs and brain^19^. In long-term research, scholars believe that *Schistosoma japonicum* and *Schistosoma mansoni* can lead to liver and intestinal damage, and *Schistosoma haematobium* mainly leads to urinary and reproductive system damage^8, 20, 21^. However, there is no report summarizing which human organs are affected by *Schistosoma japonicum* in addition to its ability to damage the digestive system. This study found that *Schistosoma* eggs are deposited in more human system organs and cause damage. *Schistosoma* eggs are mainly deposited in the digestive system (esophagus, stomach, duodenum, ileum, cecum, colon, rectum, gallbladder, pancreas, liver). In addition, a few insect eggs are also deposited in the respiratory system (lung), nervous system (brain), reproductive system (uterus, ovary, fallopian tube), but most of the case samples found that insect eggs are deposited in the lymphatic system, causing damage to the lymphatic system. Therefore, we believe that *Schistosomiasis* japonica has caused multiple organ and system damage to the human body.

A total of 1111 cases of *Schistosomiasis japonica* patients diagnosed pathologically were collected in this study, including 747 males and 364 females. Males were significantly more than females. The age of the patients ranged from 18 to 93 years old, and the main age groups were 50–59 years old (15.572%), 60–69 years old (41.855%) and 70–79 years old (27.363%). The age of onset of *Schistosomiasis* is concentrated in 60–69 years old, which is basically consistent with the age of onset of *Schistosomiasis* associated colorectal cancer^45^. The main pathological types of Schistosoma eggs were inflammation in 469 cases, adenoma in 47 cases and adenocarcinoma in 519 cases. The ages of the three pathological types were (61.555 ± 13.025) years, (63.936 ± 9.628) years and (66.730 ± 9.156) years, respectively. Inflammatory reactions play an important role in the occurrence and development of tumors^22^. In this study, according to the age sequence of the three pathological types, it is not difficult to find that the lesion may have a development trend of “Inflammation-adenoma-carcinoma”. Chronic inflammation of local tissues caused by *Schistosomiasis* may eventually lead to cancer, which is similar to the occurrence of *Schistosoma haematobium* and bladder cancer^7^, Hepatitis B Virus (HBV) infection leading to liver cancer^23^ and Helicobacter pylori (Hp) infection leading to gastric cancer^24^. We speculate that there may be a correlation between *Schistosomiasis* and cancer. At the same time, it was also found that other pathological types could be present in the lesion, including cysts, stromal tumors, malignant lymphomas and neuroendocrine tumors.

Interestingly, when analyzing the deposition sites and pathological types of *Schistosoma* eggs in this set of data, it was found that appendicitis has a high incidence in *Schistosomiasis* endemic areas, and some patients with *Schistosomiasis* appendicitis can also develop adenomas^25^. However, few literatures have reported whether *Schistosomiasis japonica* is related to appendicitis. Some chronic appendicitis occurred insidiously, and most of them have no obvious symptoms. When the appendix needs to be removed for reasons such as intestinal tumors, it is found that *Schistosoma* eggs are deposited in the appendix. It is reported that more than 30% of appendiceal specimens taken from patients in *Schistosomiasis* endemic areas can be found to be *Schistosoma* eggs^26^. The reason for this phenomenon may be that *Schistosoma* eggs are wrapped in the tissue to form a granuloma, which further fibrosis makes the appendix wall and appendix cavity narrow, prone to fecal retention and obstruction of appendix cavity.

The relationship between *Schistosoma japonicum* infection and intestinal malignancy has been controversial since the 1980s. Since then, epidemiological studies have found that people infected with *Schistosoma japonicum* have an increased risk of colorectal cancer^27–29^. But so far, no carcinogen has been isolated from adult worms or eggs of *Schistosoma japonicum*. Research has found some evidence to prove their correlation. Many literatures have reported the relationship between *Schistosomiasis japonica* and colorectal tumors, but few have reported the relationship between other tumors such as gastric cancer and small intestinal tumors and *Schistosomiasis japonica*^30–32^. In fact, in addition to causing more extensive and serious intestinal damage, *Schistosoma japonicum* infection may also have an impact on the digestive system, one of the most serious consequences is the occurrence of malignant tumors. Among many malignant tumors of digestive system, the top ten tumors with morbidity and mortality include liver cancer, gastric cancer and colorectal cancer^5^. The risk factors of gastric cancer include age, sex, race, Helicobacter pylori infection, smoking, diet, genetics and other factors^33^. At present, only a few studies believe that there may be a correlation between *Schistosomiasis* and gastric cancer, but there is no clear mechanism to confirm the relationship^34–36^. The risk factors for liver cancer include viral infection, metabolic related fatty liver disease, long-term exposure to toxicants, and parasitic infection. However, *Clonorchis sinensis* is a well-known parasite associated with liver cancer. *Schistosoma* eggs are often deposited in the hepatic portal vein, causing liver injury and causing liver and spleen *Schistosomiasis*. It is unclear whether liver and spleen *Schistosomiasis* can be used as a precancerous lesion of liver cancer^37^. Among the risk factors for colorectal cancer, those confirmed by research include age, sex, family history, intestinal inflammatory polyps, excessive alcohol consumption, smoking, obesity and long-term use of processed meat^38^. However, chronic *Schistosomiasis* as a risk factor for colorectal cancer has been debated for decades^39^. According to this group of data, the incidence of *Schistosomiasis* with adenocarcinoma is relatively high, and the most common sites are stomach, liver, colon and rectum.

This study shows that the incidence of gastric cancer in patients with gastric *Schistosomiasis* is more than 50%, and the clinical manifestations are similar to those of chronic gastritis, peptic ulcer and advanced gastric cancer, which can only be confirmed by postoperative gastroscopic biopsy or pathological examination^40^. In this study, the age of *Schistosomiasis* associated gastric cancer group was significantly higher than that of non-*Schistosomiasis* group (*P* < 0.001), and the male to female ratio of the former was much higher than that of the latter (*P* = 0.006). In terms of tumor location, gastric cancer with *Schistosomiasis* mainly occurred in the cardia, fundus and body of the stomach, which is consistent with the predilection site of gastric cancer proposed in previous reports^41^. Most non-*Schistosomiasis* gastric cancers occurred in pylorus. The difference between the two groups was statistically significant (*P* = 0.001). There were no significant differences in tumor phenotype (*P* = 0.745), differentiation (*P* = 0.232), intravascular tumor thrombus formation (*P* = 0.116) and local lymph node metastasis (*P* = 0.904) between the two groups.

The patients with non-*Schistosomiasis* liver cancer served as the control group and were compared with the *Schistosomiasis* associated liver cancer group. The two groups of liver cancer patients showed statistical differences only in the degree of tumor differentiation (*P* = 0.019), and the proportion of poorly differentiated liver cancer in the *Schistosomiasis* associated liver cancer group was higher than that in non-*Schistosomiasis* liver cancer. There were no statistically significant differences in age, gender, source of tumor cells, number of tumor cells, presence or absence of tumor thrombi in blood vessels, and integrity of tumor capsule. These data cannot clearly explain whether *Schistosomiasis* affects the occurrence and development of gastric cancer and liver cancer. However, *Schistosomiasis* associated gastric cancer and *Schistosomiasis* associated liver cancer differ from non-*Schistosomiasis* tumors in age, sex, susceptible site and tumor differentiation, which means that *Schistosomiasis* may change the relevant mechanism of tumorigenesis.

In recent years, some studies have shown that the occurrence of colon cancer is related to the infection of some pathogens, such as bacteria^42, 43^, parasites^38, 44^ and other pathogens. In this study, we found statistically significant differences in age and sex between the *Schistosomiasis* associated colon cancer group and the non-*Schistosomiasis* group. The average age of patients in the *Schistosomiasis* associated colon cancer group was (67.331 ± 10.082) years, which was significantly higher than that in the non-*Schistosomiasis* group (61.194 ± 11.675) years, and the difference was statistically significant (*P* < 0.001). The male to female ratio of *Schistosomiasis* associated colon cancer group was higher than that of non-*Schistosomiasis* group (*P* = 0.005). There was significant difference in tumor differentiation between the two groups (*P* = 0.013). There was no difference in tumor location, tumor morphology, tumor thrombus and local lymph node metastasis between the two groups. Our results showed that in addition to rectal cancer, there were chronic inflammation and adenomas in the colonic lesions of *Schistosomiasis*. It is known that patients with long-term *Schistosomiasis* associated chronic inflammation and adenomas have an increased risk of colorectal cancer^45^. These pathological changes are considered to be the pathological basis of the malignant potential of *Schistosomiasis* associated colorectal cancer.

Among rectal cancer patients, there are statistical differences in age and gender between the *Schistosomiasis* associated rectal cancer group and the non-*Schistosomiasis* group. The average age of the former is (65.706 ± 9.5549) years, which is higher than the latter (59.517 ± 15.242) years, and the difference is statistically significant (*P* < 0.001). There were more males than females in both groups, and the male to female ratio in the former was significantly higher than that in the non-*Schistosomiasis* group (*P* = 0.005). There was a significant difference in tumor differentiation between the two groups of patients (*P* = 0.013). There was no significant difference in tumor morphology, tumor thrombus, and local lymph node metastasis between the two groups. Low differentiated tumors often have poor prognosis, which is consistent with the reported poor prognosis of *Schistosomiasis* associated rectal cancer compared to non-*Schistosomiasis* rectal cancer in the literature. Moreover, regardless of the intervention of treatment methods, the prognosis of the former is relatively poor compared to the latter^29, 46^.

## Conclusion

In summary, *Schistosoma japonicum* can cause damage to multiple systems and organs, and the types of lesions are diverse. Inflammation, adenoma and carcinoma are still common lesion types. According to the average age of the three lesion types, the pathological change process showed a trend of “Inflammation-adenoma-carcinoma”. So, we speculate that *Schistosomiasis* may be associated with the occurrence of cancer. Some pathological characteristics of patients with *Schistosomiasis* associated tumors are different from those of non-*Schistosomiasis* associated tumors. *Schistosomiasis* may affect the pathogenesis of tumors. Further exploration of the correlation between *Schistosomiasis* and digestive system tumors and the pathogenesis of *Schistosomiasis* associated tumors will help to prevent and treat malignant tumors, reduce the incidence of malignant tumors in endemic areas, and improve the survival time of patients with chronic *Schistosomiasis*.

## Data Availability

The data were obtained in Department of infectious diseases, the First Affiliated Hospital of Wannan Medical College and Chizhou first people’s Hospital. The datasets used and during the current study are available from the corresponding author on reasonable request.
